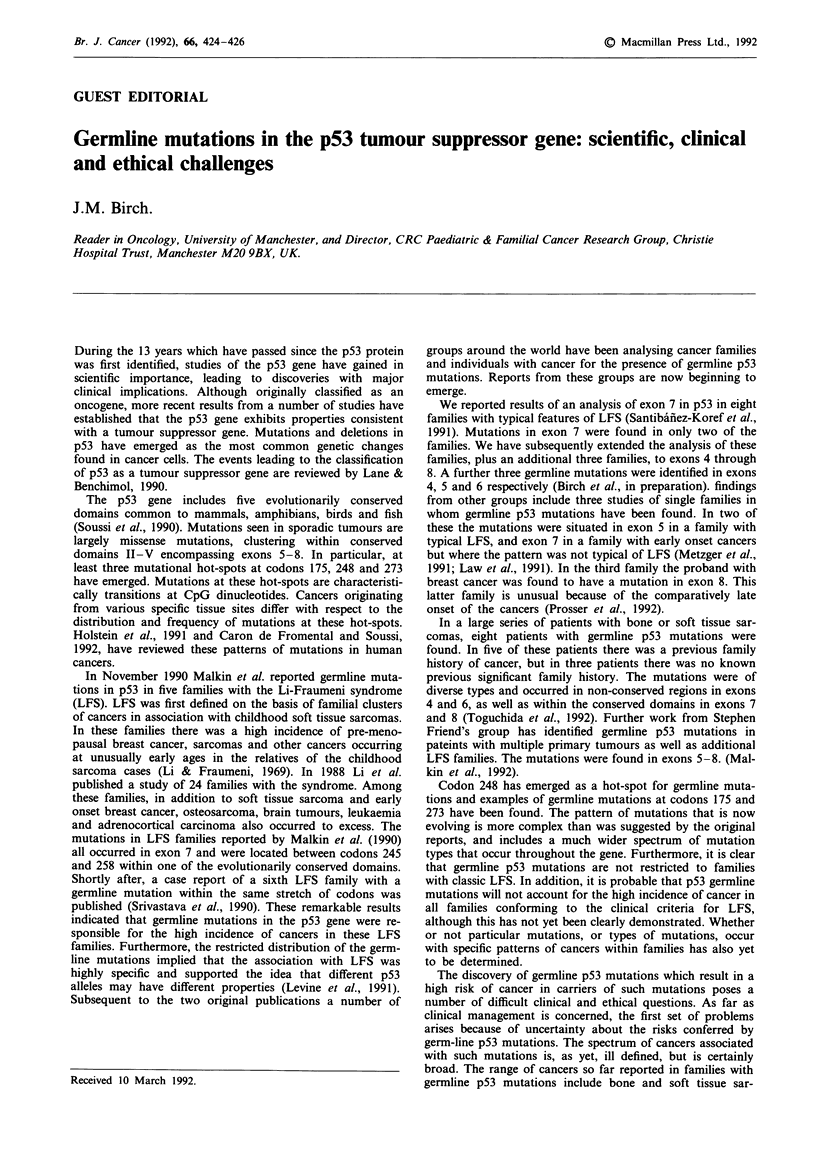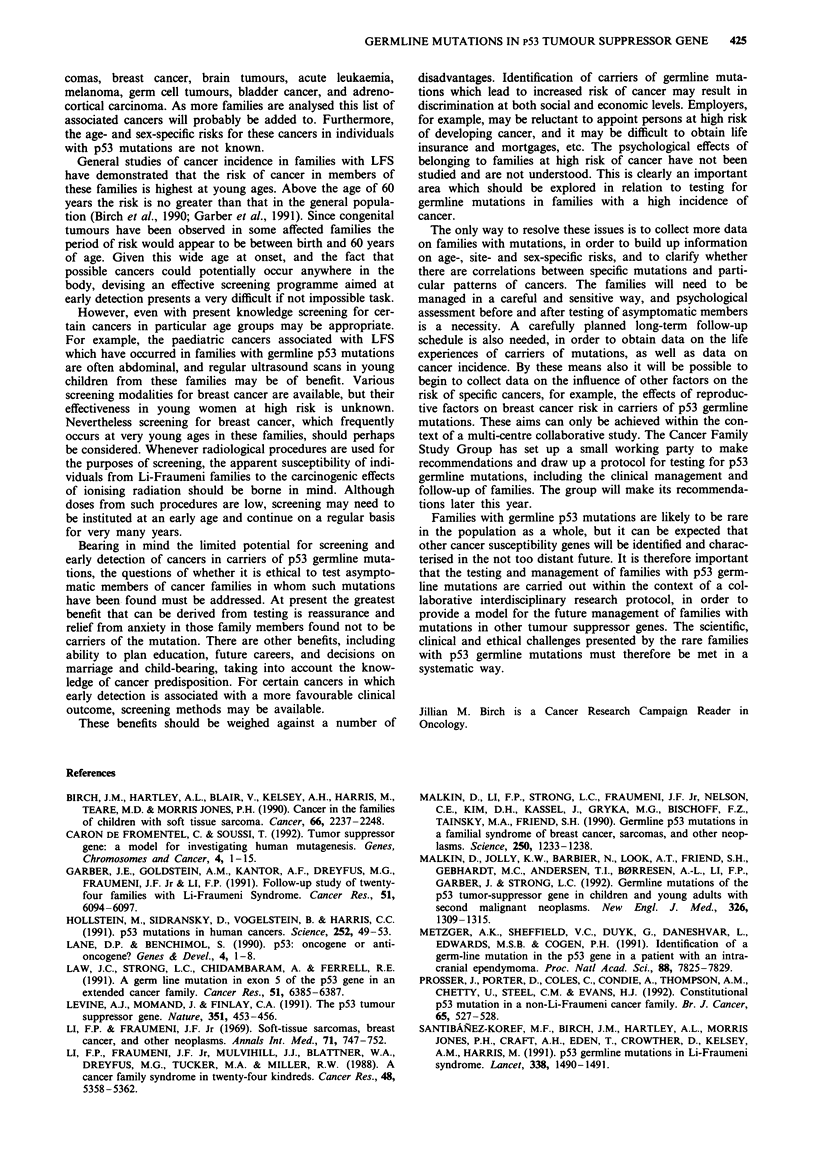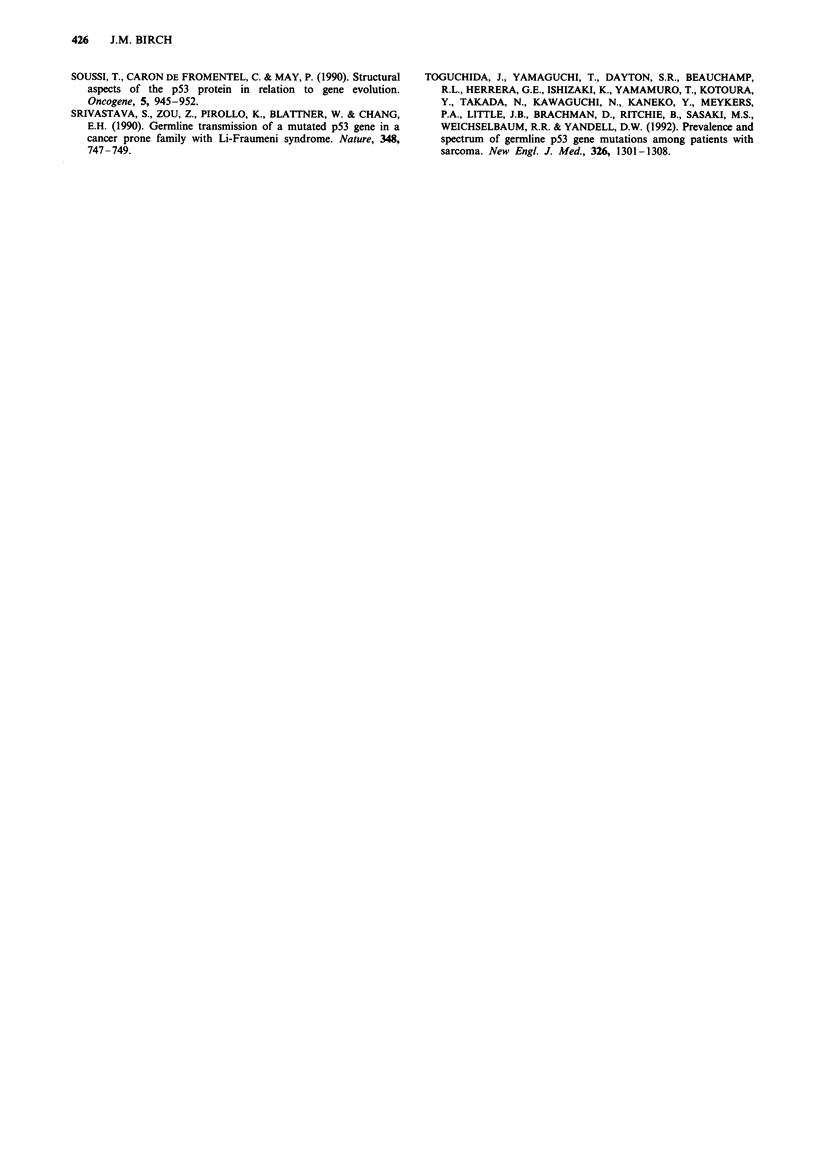# Germline mutations in the p53 tumour suppressor gene: scientific, clinical and ethical challenges.

**DOI:** 10.1038/bjc.1992.290

**Published:** 1992-09

**Authors:** J. M. Birch


Br. J. Cancer (1992), 66, 424–426 © Macmillan Press Ltd., 1992

GUEST EDITORIAL

### Germline mutations in the p53 tumour suppressor gene: scientific, clinical and ethical challenges

J.M. Birch.

*Reader in Oncology, University of Manchester, and Director, CRC Paediatric & Familial Cancer Research Group, Christie Hospital Trust, Manchester M20 9BX, UK.*

During the 13 years which have passed since the p53 protein was first identified, studies of the p53 gene have gained in scientific importance, leading to discoveries with major clinical implications. Although originally classified as an oncogene, more recent results from a number of studies have established that the p53 gene exhibits properties consistent with a tumour suppressor gene. Mutations and deletions in p53 have emerged as the most common genetic changes found in cancer cells. The events leading to the classification of p53 as a tumour suppressor gene are reviewed by Lane & Benchimol, 1990.

The p53 gene includes five evolutionarily conserved domains common to mammals, amphibians, birds and fish (Soussi *et al.*, 1990). Mutations seen in sporadic tumours are largely missense mutations, clustering within conserved domains II–V encompassing exons 5–8. In particular, at least three mutational hot-spots at codons 175, 248 and 273 have emerged. Mutations at these hot-spots are characteristically transitions at CpG dinucleotides. Cancers originating from various specific tissue sites differ with respect to the distribution and frequency of mutations at these hot-spots. Holstein *et al.*, 1991 and Caron de Fromental and Soussi, 1992, have reviewed these patterns of mutations in human cancers.

In November 1990 Malkin *et al.* reported germline mutations in p53 in five families with the Li-Fraumeni syndrome (LFS). LFS was first defined on the basis of familial clusters of cancers in association with childhood soft tissue sarcomas. In these families there was a high incidence of pre-menopausal breast cancer, sarcomas and other cancers occurring at unusually early ages in the relatives of the childhood sarcoma cases (Li & Fraumeni, 1969). In 1988 Li *et al.* published a study of 24 families with the syndrome. Among these families, in addition to soft tissue sarcoma and early onset breast cancer, osteosarcoma, brain tumours, leukaemia and adrenocortical carcinoma also occurred to excess. The mutations in LFS families reported by Malkin *et al.* (1990) all occurred in exon 7 and were located between codons 245 and 258 within one of the evolutionarily conserved domains. Shortly after, a case report of a sixth LFS family with a germline mutation within the same stretch of codons was published (Srivastava *et al.*, 1990). These remarkable results indicated that germline mutations in the p53 gene were responsible for the high incidence of cancers in these LFS families. Furthermore, the restricted distribution of the germline mutations implied that the association with LFS was highly specific and supported the idea that different p53 alleles may have different properties (Levine *et al.*, 1991). Subsequent to the two original publications a number of groups around the world have been analysing cancer families and individuals with cancer for the presence of germline p53 mutations. Reports from these groups are now beginning to emerge.

We reported results of an analysis of exon 7 in p53 in eight families with typical features of LFS (Santibáñez-Koref *et al.*, 1991). Mutations in exon 7 were found in only two of the families. We have subsequently extended the analysis of these families, plus an additional three families, to exons 4 through 8. A further three germline mutations were identified in exons 4, 5 and 6 respectively (Birch *et al.*, in preparation). findings from other groups include three studies of single families in whom germline p53 mutations have been found. In two of these the mutations were situated in exon 5 in a family with typical LFS, and exon 7 in a family with early onset cancers but where the pattern was not typical of LFS (Metzger *et al.*, 1991; Law *et al.*, 1991). In the third family the proband with breast cancer was found to have a mutation in exon 8. This latter family is unusual because of the comparatively late onset of the cancers (Prosser *et al.*, 1992).

In a large series of patients with bone or soft tissue sarcomas, eight patients with germline p53 mutations were found. In five of these patients there was a previous family history of cancer, but in three patients there was no known previous significant family history. The mutations were of diverse types and occurred in non-conserved regions in exons 4 and 6, as well as within the conserved domains in exons 7 and 8 (Toguchida *et al.*, 1992). Further work from Stephen Friend's group has identified germline p53 mutations in pateints with multiple primary tumours as well as additional LFS families. The mutations were found in exons 5–8. (Malkin *et al.*, 1992).

Codon 248 has emerged as a hot-spot for germline mutations and examples of germline mutations at codons 175 and 273 have been found. The pattern of mutations that is now evolving is more complex than was suggested by the original reports, and includes a much wider spectrum of mutation types that occur throughout the gene. Furthermore, it is clear that germline p53 mutations are not restricted to families with classic LFS. In addition, it is probable that p53 germline mutations will not account for the high incidence of cancer in all families conforming to the clinical criteria for LFS, although this has not yet been clearly demonstrated. Whether or not particular mutations, or types of mutations, occur with specific patterns of cancers within families has also yet to be determined.

The discovery of germline p53 mutations which result in a high risk of cancer in carriers of such mutations poses a number of difficult clinical and ethical questions. As far as clinical management is concerned, the first set of problems arises because of uncertainty about the risks conferred by germ-line p53 mutations. The spectrum of cancers associated with such mutations is, as yet, ill defined, but is certainly broad. The range of cancers so far reported in families with germline p53 mutations include bone and soft tissue sar-

Received 10 March 1992.

comas, breast cancer, brain tumours, acute leukaemia, melanoma, germ cell tumours, bladder cancer, and adrenocortical carcinoma. As more families are analysed this list of associated cancers will probably be added to. Furthermore, the age- and sex-specific risks for these cancers in individuals with p53 mutations are not known.

General studies of cancer incidence in families with LFS have demonstrated that the risk of cancer in members of these families is highest at young ages. Above the age of 60 years the risk is no greater than that in the general population (Birch *et al.*, 1990; Garber *et al.*, 1991). Since congenital tumours have been observed in some affected families the period of risk would appear to be between birth and 60 years of age. Given this wide age at onset, and the fact that possible cancers could potentially occur anywhere in the body, devising an effective screening programme aimed at early detection presents a very difficult if not impossible task.

However, even with present knowledge screening for certain cancers in particular age groups may be appropriate. For example, the paediatric cancers associated with LFS which have occurred in families with germline p53 mutations are often abdominal, and regular ultrasound scans in young children from these families may be of benefit. Various screening modalities for breast cancer are available, but their effectiveness in young women at high risk is unknown. Nevertheless screening for breast cancer, which frequently occurs at very young ages in these families, should perhaps be considered. Whenever radiological procedures are used for the purposes of screening, the apparent susceptibility of individuals from Li-Fraumeni families to the carcinogenic effects of ionising radiation should be borne in mind. Although doses from such procedures are low, screening may need to be instituted at an early age and continue on a regular basis for very many years.

Bearing in mind the limited potential for screening and early detection of cancers in carriers of p53 germline mutations, the questions of whether it is ethical to test asymptomatic members of cancer families in whom such mutations have been found must be addressed. At present the greatest benefit that can be derived from testing is reassurance and relief from anxiety in those family members found not to be carriers of the mutation. There are other benefits, including ability to plan education, future careers, and decisions on marriage and child-bearing, taking into account the knowledge of cancer predisposition. For certain cancers in which early detection is associated with a more favourable clinical outcome, screening methods may be available.

These benefits should be weighed against a number of disadvantages. Identification of carriers of germline mutations which lead to increased risk of cancer may result in discrimination at both social and economic levels. Employers, for example, may be reluctant to appoint persons at high risk of developing cancer, and it may be difficult to obtain life insurance and mortgages, etc. The psychological effects of belonging to families at high risk of cancer have not been studied and are not understood. This is clearly an important area which should be explored in relation to testing for germline mutations in families with a high incidence of cancer.

The only way to resolve these issues is to collect more data on families with mutations, in order to build up information on age-, site- and sex-specific risks, and to clarify whether there are correlations between specific mutations and particular patterns of cancers. The families will need to be managed in a careful and sensitive way, and psychological assessment before and after testing of asymptomatic members is a necessity. A carefully planned long-term follow-up schedule is also needed, in order to obtain data on the life experiences of carriers of mutations, as well as data on cancer incidence. By these means also it will be possible to begin to collect data on the influence of other factors on the risk of specific cancers, for example, the effects of reproductive factors on breast cancer risk in carriers of p53 germline mutations. These aims can only be achieved within the context of a multi-centre collaborative study. The Cancer Family Study Group has set up a small working party to make recommendations and draw up a protocol for testing for p53 germline mutations, including the clinical management and follow-up of families. The group will make its recommendations later this year.

Families with germline p53 mutations are likely to be rare in the population as a whole, but it can be expected that other cancer susceptibility genes will be identified and characterised in the not too distant future. It is therefore important that the testing and management of families with p53 germline mutations are carried out within the context of a collaborative interdisciplinary research protocol, in order to provide a model for the future management of families with mutations in other tumour suppressor genes. The scientific, clinical and ethical challenges presented by the rare families with p53 germline mutations must therefore be met in a systematic way.

Jillian M. Birch is a Cancer Research Campaign Reader in Oncology.

**References**

BIRCH, J.M., HARTLEY, A.L., BLAIR, V., KELSEY, A.H., HARRIS, M., TEARE, M.D. & MORRIS JONES, P.H. (1990). Cancer in the families of children with soft tissue sarcoma. *Cancer*, **66**, 2237–2248.

CARON DE FROMENTEL, C. & SOUSSI, T. (1992). Tumor suppressor gene: a model for investigating human mutagenesis. *Genes, Chromosomes and Cancer*, **4**, 1–15.

GARBER, J.E., GOLDSTEIN, A.M., KANTOR, A.F., DREYFUS, M.G., FRAUMENI, J.F. Jr & LI, F.P. (1991). Follow-up study of twenty-four families with Li-Fraumeni Syndrome. *Cancer Res.*, **51**, 6094–6097.

HOLLSTEIN, M., SIDRANSKY, D., VOGELSTEIN, B. & HARRIS, C.C. (1991). p53 mutations in human cancers. *Science*, **252**, 49–53.

LANE, D.P. & BENCHIMOL, S. (1990). p53: oncogene or anti-oncogene? *Genes & Devel.*, **4**, 1–8.

LAW, J.C., STRONG, L.C., CHIDAMBARAM, A. & FERRELL, R.E. (1991). A germ line mutation in exon 5 of the p53 gene in an extended cancer family. *Cancer Res.*, **51**, 6385–6387.

LEVINE, A.J., MOMAND, J. & FINLAY, C.A. (1991). The p53 tumour suppressor gene. *Nature*, **351**, 453–456.

LI, F.P. & FRAUMENI, J.F. Jr (1969). Soft-tissue sarcomas, breast cancer, and other neoplasms. *Annals Int. Med.*, **71**, 747–752.

LI, F.P., FRAUMENI, J.F. Jr, MULVIHILL, J.J., BLATTNER, W.A., DREYFUS, M.G., TUCKER, M.A. & MILLER, R.W. (1988). A cancer family syndrome in twenty-four kindreds. *Cancer Res.*, **48**, 5358–5362.

MALKIN, D., LI, F.P., STRONG, L.C., FRAUMENI, J.F. Jr, NELSON, C.E., KIM, D.H., KASSEL, J., GRYKA, M.G., BISCHOFF, F.Z., TAINSKY, M.A., FRIEND, S.H. (1990). Germline p53 mutations in a familial syndrome of breast cancer, sarcomas, and other neoplasms. *Science*, **250**, 1233–1238.

MALKIN, D., JOLLY, K.W., BARBIER, N., LOOK, A.T., FRIEND, S.H., GEBHARDT, M.C., ANDERSEN, T.I., BØRRESEN, A.-L., LI, F.P., GARBER, J. & STRONG, L.C. (1992). Germline mutations of the p53 tumor-suppressor gene in children and young adults with second malignant neoplasms. *New Engl. J. Med.*, **326**, 1309–1315.

METZGER, A.K., SHEFFIELD, V.C., DUYK, G., DANESHVAR, L., EDWARDS, M.S.B. & COGEN, P.H. (1991). Identification of a germ-line mutation in the p53 gene in a patient with an intracranial ependymoma. *Proc. Natl Acad. Sci.*, **88**, 7825–7829.

PROSSER, J., PORTER, D., COLES, C., CONDIE, A., THOMPSON, A.M., CHETTY, U., STEEL, C.M. & EVANS, H.J. (1992). Constitutional p53 mutation in a non-Li-Fraumeni cancer family. *Br. J. Cancer*, **65**, 527–528.

SANTIBÁÑEZ-KOREF, M.F., BIRCH, J.M., HARTLEY, A.L., MORRIS JONES, P.H., CRAFT, A.H., EDEN, T., CROWTHER, D., KELSEY, A.M., HARRIS, M. (1991). p53 germline mutations in Li-Fraumeni syndrome. *Lancet*, **338**, 1490–1491.

SOUSSI, T., CARON DE FROMENTEL, C. & MAY, P. (1990). Structural aspects of the p53 protein in relation to gene evolution. *Oncogene*, **5**, 945–952.

SRIVASTAVA, S., ZOU, Z., PIROLLO, K., BLATTNER, W. & CHANG, E.H. (1990). Germline transmission of a mutated p53 gene in a cancer prone family with Li-Fraumeni syndrome. *Nature*, **348**, 747–749.

TOGUCHIDA, J., YAMAGUCHI, T., DAYTON, S.R., BEAUCHAMP, R.L., HERRERA, G.E., ISHIZAKI, K., YAMAMURO, T., KOTOURA, Y., TAKADA, N., KAWAGUCHI, N., KANEKO, Y., MEYKERS, P.A., LITTLE, J.B., BRACHMAN, D., RITCHIE, B., SASAKI, M.S., WEICHSELBAUM, R.R. & YANDELL, D.W. (1992). Prevalence and spectrum of germline p53 gene mutations among patients with sarcoma. *New Engl. J. Med.*, **326**, 1301–1308.